# 18F-FDG PET/CT Imaging of Extranodal Rosai-Dorfman Disease with Hepatopancreatic Involvement - A Pictorial and Literature Review

**DOI:** 10.7759/cureus.392

**Published:** 2015-12-03

**Authors:** Faiq Shaikh, Omer Awan, Sohaib Mohiuddin, Saleem Farooqui, Salman A Khan, William McCartney

**Affiliations:** 1 Imaging Informatics, University of Pittsburgh Medical Center, Pittsburgh, PA.; 2 Molecular Imaging, Cellsight Technologies, Inc., San Francisco, CA.; 3 Department of Radiology, Dartmouth Hitchcock Medical Center; 4 Department of Radiology, Kansas University Medical Center, Wichita, KS.; 5 Department of Radiology, St Agnes Hospital, Baltimore, MD; 6 Department of Internal Medicine, University of Missouri-Kansas City; 7 Department of Radiology, University of North Carolina Hospitals, Chapel Hill, NC.

**Keywords:** fdg pet/ct, hepatopancreatic, extranodal, rosai-dorfman disease

## Abstract

We share our experience with serial PET/CT imaging on a patient with extranodal Rosai-Dorfman disease (RDD) with hepatopancreatic involvement. RDD is a benign proliferative disorder of histiocytes mainly involving the lymph nodes. It typically presents with fever and painless cervical lymphadenopathy in young adults and less than half of RDS cases demonstrate extranodal involvement. RDD involvement of the liver and pancreas is extremely rare, and this case highlights the role of PET/CT in its management.

## Introduction

Rosai-Dorfman syndrome (RDS) is a rare histiocytic proliferative disorder commonly involving the lymph nodes and presenting with non-specific symptoms, such as fever and elevated erythrocyte sedimentation rate (ESR). Less than half of the cases demonstrate extranodal involvement, with symptomology reflecting the afflicted organ-systems. Hepatopancreatic involvement of RDD is extremely rare, and our case highlights the role of FDG PET/CT in its detection of management.

## Case presentation

A 59-year-old female presented with abdominal pain, several episodes of syncope, and uncontrolled hyperglycemia. Her initial workup revealed findings suggestive of acute pancreatitis, and initial imaging was suggestive of a pancreatic mass. Informed patient consent was obtained for her treatment. She was taken to the operating room for a Whipple's procedure, and the surgical pathology revealed Rosai-Dorfman disease.

**Figure 1:**
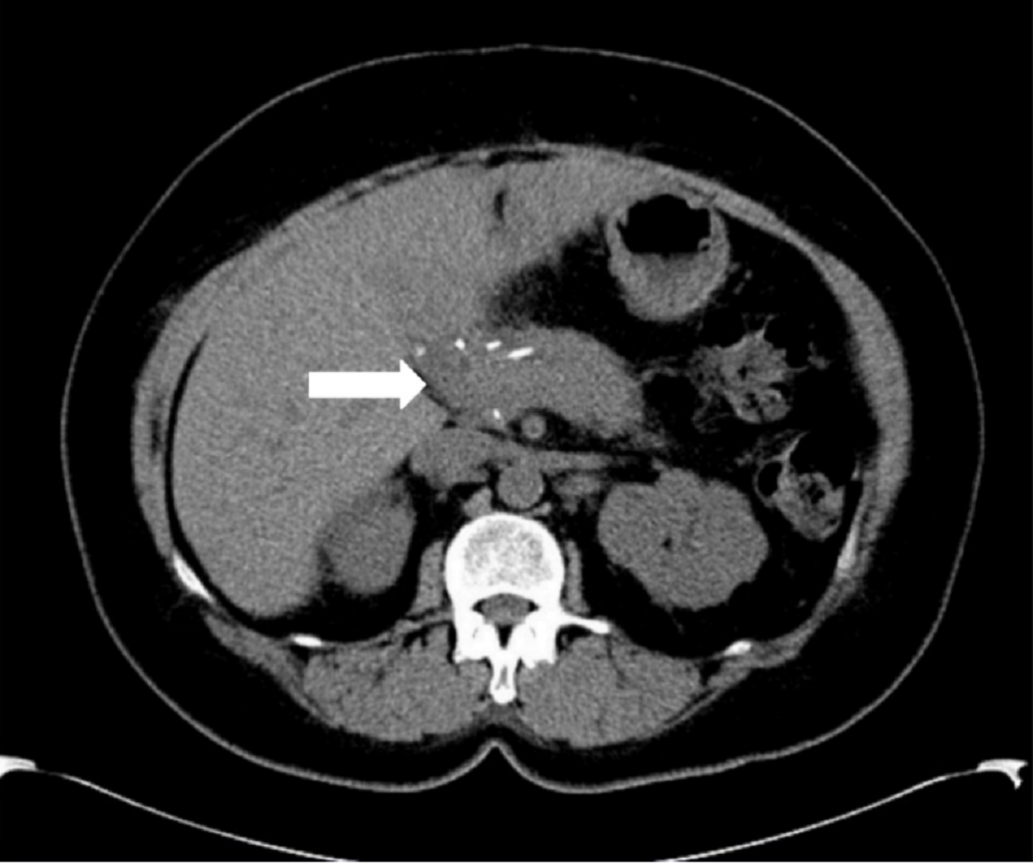
Postoperative CT scan The non-contrast enhanced CT axial image demonstrates post-Whipple changes and a hypodense lesion in the pancreatic body/tail remnant (white arrow).

**Figure 2:**
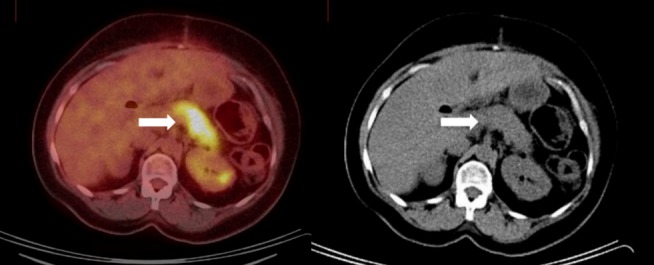
Postoperative initial PET/CT scan There is an FDG-avid lesion in the pancreatic tail on the PET axial image *(white arrow, left image) *with a corresponding hypodense correlate on the non-contrast enhanced CT axial image acquired as part of the same study *(white arrow, right image).*

A postoperative computed tomography (CT) scan was performed, which demonstrated no evidence of extrapancreatic involvement or lymphadenopathy (Figure 1). At that time, an 18F-FDG PET/CT scan was performed to look for evidence of inflammation within the active RDD lesions to help determine the need for steroid administration. The scan demonstrated intensely increased FDG uptake in the body and tail of the pancreas (Figure 2), suggestive of the active inflammatory process associated with RDD.

**Figure 3:**
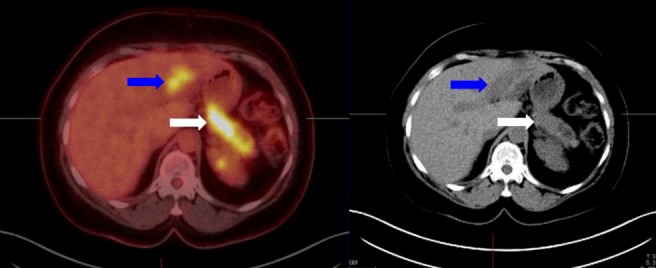
Follow-up PET/CT scan There is a new FDG-avid lesion in the left hepatic lobe on the PET axial images *(blue arrow, left image) *with a corresponding hypodense correlate on the CT axial image *(blue arrow, left image)*. Also seen again the original FDG-avid pancreatic tail lesion *(white arrow).*

High-dose intravenous steroid therapy was initiated, and the patient was brought back for follow-up. A subsequent PET/CT was performed, which demonstrated a new focus of increased FDG uptake in the left hepatic lobe, in addition to the known pancreatic body/tail lesion (Figure 3). This was suggestive of the failure of steroid therapy based on the interval progression of RDD to multivisceral (hepatopancreatic) involvement. This led to the consideration of imatinib mesylate (Gleevec) as a potential second-line therapeutic option. However, further follow-up for the case was not available.

## Discussion

Rosai-Dorfman disease (RDD) is a rare, benign histiocytic proliferative disorder of unknown etiology [1]. It commonly occurs during the first or second decade of life and is usually characterized by massive painless lymphadenopathy and other systemic manifestations, including fever, night sweats, and weight loss. The accompanying laboratory findings include leukocytosis with neutrophilia, elevated erythrocyte sedimentation rate, and polyclonal hypergammaglobulinemia. Extranodal involvement has been noted in more than 40% of cases, and cutaneous lesions represent the most common form of the extranodal disease [2]. There have been a few reports of extranodal RDD involving the nervous system, heart, and other organ systems [3-4].

Cutaneous RDD typically presents with papules and plaques that can grow to form nodules with satellite lesions that resolve into fibrotic plaques before spontaneous regression [5]. Histologically, RDD is characterized by dense nodular infiltrates of large polygonal histiocytes called Rosai-Dorfman cells demonstrating emperipolesis, a phenomenon whereby inflammatory cells, such as lymphocytes and plasma cells, reside intact within the abundant cytoplasm of histiocytes. These cells demonstrate indistinct borders and large vesicular nuclei with prominent nucleoli and display positive staining for CD68 and S-100, and negative staining for CD1a on immunohistochemistry [6]. The differential diagnosis of RDD typically includes other histiocytic and lymphoproliferative diseases, such as Langerhans’ cell histiocytosis, which differs from RDD in terms of being a systemic disease with a proliferation of positive to S100, CD1a, and langherin histiocytes, combined with an intense inflammatory infiltrate, but without the evidence of emperipolesis [7-9].

Gastrointestinal localization of RDD, especially in the liver and pancreas, is extremely rare. Typically, the liver is affected as a part of the systematic spread of RDD with nodal and wide extranodal involvement as demonstrated by Lauwers et al. [7-8]. Pancreatic involvement with RDD may be characterized by nonspecific symptoms, such as abdominal or back pain, and endocrine insufficiency, such as hyperglycemia that was reported in this case.

Because the etiology of RDD is not well understood, the treatment is still nonspecific and controversial. Treatment is recommended only in patients who are symptomatic or have vital organ or systemic involvement, as RDD is reported to be self-limited in approximately 20% of the cases [9]. Complete surgical resection is the best option for treatment of the localized RDD, while steroids are the first-line therapeutic option for symptomatic cases with extensive organ-systemic involvement [9]. For complicated cases demonstrating orbit, airway, or central nervous system involvement, radiotherapy may be attempted [10]. Chemotherapy has been attempted, with varying degrees of success, for disseminated RDD or cases refractory to surgery or other modalities [11]. Given the suggestion of active inflammatory process in this case, based on the high ESR on lab findings and FDG avidity on PET imaging, intravenous steroid therapy was initiated. 

Like various other lymphoproliferative disorders, benign and malignant alike, RDD lesions have been shown to be FDG-avid [12]. There have been reports of FDG avidity in the extranodal areas of involvement as well, such as the skeletal system [13]. There is at least one case report that demonstrated resolution of FDG avidity in RD lesions in response to therapy [14]. FDG-avidity of RDD lesions is attributable to the intense glucose-dependence of the proliferating histiocytes as well as the infiltrating inflammatory cells. The PET/CT performed on our patient demonstrated intensely increased uptake in a poorly circumscribed region in the pancreatic body/tail (on initial PET), intervally progressing to involve another site within the left hepatic lobe (on follow-up PET).* *The findings of the latter were suggestive of failure of the steroid therapy to regress or halt the progression of the disease.

## Conclusions

This case demonstrates the role of PET-CT imaging in the detection of RDD with visceral involvement based on the FDG avidity attributable to the infiltrative and inflammatory component of the disease process. Furthermore, it suggests the usefulness of this modality in the assessment for disease progression to other extranodal sites when used for therapy response assessment.
